# The direction of cross obesity after puberty in male but not female offspring

**DOI:** 10.1186/s12864-015-2164-2

**Published:** 2015-11-06

**Authors:** Stefan Kärst, Danny Arends, Sebastian Heise, Jan Trost, Marie-Laure Yaspo, Vyacheslav Amstislavskiy, Thomas Risch, Hans Lehrach, Gudrun A. Brockmann

**Affiliations:** Albrecht Daniel Thaer-Institut für Agrar- und Gartenbauwissenschaften, Humboldt-Universität zu Berlin, Invalidenstraße 42, D-10115 Berlin, Germany; Max Planck Institute for Molecular Genetics, Gene Regulation and Systems Biology of Cancer, Ihnestraße 63-73, 14195 Berlin, Germany

**Keywords:** Allele-specific gene expression, Allelic imbalance, High fat diet, Imprinting, Peg3, Circadian rhythm, Sex differences, Sex hormones

## Abstract

**Background:**

We investigated parent-of-origin and allele-specific expression effects on obesity and hepatic gene expression in reciprocal crosses between the Berlin Fat Mouse Inbred line (BFMI) and C57Bl/6NCrl (B6N).

**Results:**

We found that F1-males with a BFMI mother developed 1.8 times more fat mass on a high fat diet at 10 weeks than F1-males of a BFMI father. The phenotype was detectable from six weeks on and was preserved after cross-fostering. RNA-seq data of liver provided evidence for higher biosynthesis and elongation of fatty acids (*p* = 0.00635) in obese male offspring of a BFMI mother versus lean offspring of a BFMI father. Furthermore, fatty acid degradation (*p* = 0.00198) and the peroxisome pathway were impaired (*p* = 0.00094). The circadian rhythm was affected as well (*p* = 0.00087). Among the highest up-regulated protein coding genes in obese males were Acot4 (1.82 fold, *p* = 0.022), Cyp4a10 (1.35 fold, *p* = 0.026) and Cyp4a14 (1.32 fold, *p* = 0.012), which hydroxylize fatty acids and which are known to be increased in liver steatosis. Obese males showed lower expression of the genetically imprinted and paternally expressed 3 (Peg3) gene (0.31 fold, *p* = 0.046) and higher expression of the androgen receptor (Ar) gene (2.38 fold, *p* = 0.068). Allelic imbalance was found for expression of ATP-binding cassette transporter gene Abca8b. Several of the differentially expressed genes contain estrogen response elements.

**Conclusions:**

Parent-of-origin effects during gametogenesis and/or fetal development in an obese mother epigenetically modify the transcription of genes that lead to enhanced fatty acid synthesis and impair β-oxidation in the liver of male, but not female F1 offspring. Down-regulation of Peg3 could contribute to trigger this metabolic setting. At puberty, higher amounts of the androgen receptor and altered access to estrogen response elements in affected genes are likely responsible for male specific expression of genes that were epigenetically triggered. A suggestive lack of estrogen binding motifs was found for highly down-regulated genes in adult hepatocytes of obese F1 males (*p* = 0.074).

**Electronic supplementary material:**

The online version of this article (doi:10.1186/s12864-015-2164-2) contains supplementary material, which is available to authorized users.

## Background

Besides nutrition and sedentary lifestyle, genetic predisposition contributes to obesity. To assess the genetic determinants of obesity, many large-scale genome-wide association studies were performed in different human populations. These studies led to the identification of about 350 genomic loci contributing to obesity [1]. However, their effects are small and the overall contribution of all detected loci to the variance in a population was small (0.01 % to 0.34 %) [2–6]. Therefore, additional factors could contribute to obesity in an individual. Missing heritability can be hidden in genetic effects of rare alleles, epistatic interaction and gene-by-environment interaction, but can be also contributed to by parent-of-origin specific effects. Parent-of-origin effects include the Y chromosome, mitochondria, parental-specific genetic imprinting, and maternal environmental effects during pregnancy and the suckling period.

In a mouse model of juvenile obesity, we tested, if parental obesity influences the transmission of predisposition for adiposity to F1 offspring. We used the Berlin Fat Mouse Inbred line (BFMI) which carries a recessive mutation responsible for high fat deposition in particular during early development between four and ten weeks [7]. At ten weeks BFMI males are 5.5 times as obese as C57BL/6Nrl mice (B6N).

In reciprocal crosses between BFMI and B6N mice we tested the development of obesity in F1 males and females and their response to the obese parent with respect to gene expression in the liver. The results provide evidence for regulation of genes by the influence of the direction-of-cross, which leads to obesity and fatty liver exclusively in male offspring of obese BFMI mothers.

## Methods

### Animals

Reciprocal crosses were generated from the Berlin Fat Mouse Inbred line BFMI860-12/Hber (BFMI) and C57BL/6NCrl (B6N). Seven BFMI males were mated with seven B6N females and six B6N males with six BFMI860 females to generate 48 F1 animals (deemed patBFMI and matBFMI, respectively). Each reciprocal F1 offspring groups consisted of 12 males and 12 females. From these animals three F1 males (matBFMI) from three BFMI females and three F1 males (patBFMI) from three B6N females were used for RNA-seq transcriptome measurements. One high fat diet raised male and female from each inbred parental strain (BFMI and B6N) were used as parental strain control animals and measured via RNA-seq as well. For the cross-fostering experiment, six BFMIxB6N and six B6NxBFMI crosses generated two additional groups of reciprocal F1 animals with a total of 88 animals, 46 with a BFMI father (23 males, 23 females) and 42 with a BFMI mother (24 males, 18 females). For cross-fostering, litters were exchanged between B6N and BFMI mothers within 12 hours after birth. The litter size ranged between four and eleven offspring. Two animals per cross-fostering group were excluded due to outlier testing (R-package “outliers”).

Parents were fed a standard breeding diet (SBD). F1-animals were fed a high fat diet (HFD) from weaning at 21 days until 70 days when offspring was euthanized and dissected. The SBD contained 16.7 MJ/kg of metabolisable energy, 11 % from fat, 36 % from proteins and 53 % from carbohydrates (V1534-000, ssniff EF R/M, Ssniff GmbH, Soest/Germany). The HFD contained 19.5 MJ/kg of metabolisable energy, 45 % from fat, 24 % from proteins and 31 % from carbohydrates (E15103–34, ssniff EF R/M, Ssniff GmbH, Soest/Germany). All experimental treatments of animals were approved by the German Animal Welfare Authorities (approval no. G016/11).

### Phenotyping

Body weight and body composition (total lean mass, total fat mass) were recorded weekly from week three and four. Body composition was measured using EchoMRI whole-body composition analyzer (Echo Medical Systems, Houston, TX, USA). At ten weeks blood glucose levels were measured prior to dissection after 2 hours of fasting using a glucometer (Contour, Bayer, Leverkusen, Germany). Liver triglycerides and proteins were measured in homogenized liver samples by commercially available kits (DC Protein Assay, Bio-Rad, München, Germany; TRIGS Triglycerides Assay, Randox, Crumlin, United Kingdom). For test statistics between the matBFMI and patBFMI, we applied a linear model using anova in R. Since litter size had a significant effect on all traits, phenotypic values were adjusted for litter size before statistic tests were performed, except for blood glucose and liver triglycerides.

### RNA preparation and sequencing

RNA sequencing was performed on 10 liver samples: 1 male and 1 female B6N mouse, 1 male and 1 female BFMI mouse, 3 F1 (BFMI x B6N) males (patBFMI), and 3 F1 (B6N x BFMI) males (matBFMI). The F1 males were of non-cross-fostered mice. Tissue samples were frozen in liquid nitrogen and stored at –80 °C until RNA preparation. RNA was prepared using the RiboZero RNA library protocol. Paired end RNA sequencing was run on an Illumina HiSeq2000 machine.

### Analysis of RNA-Seq data

After sequencing, bcl2fastq v1.8.4 [8] was used for base calling and demultiplexing. Paired end reads were trimmed using Trimmomatic [9] using standard settings. TruSeq3-PE adapter sequences provided by Illumina were used for adapter trimming. Reads were aligned against Mus musculus GRCm38 reference genome (Ensembl) using BWA [10], after which GATK [11] was applied to do base quality score recalibration, indel realignment, duplicate removal, SNP/INDEL discovery and genotyping across all 10 samples using standard hard filtering parameters or variant quality score recalibration according to GATK Best Practices recommendations [12].

Raw reads from RNA-Seq data were extracted from BAM files using the GenomicFeatures package using the Mus_musculus.GRCm38.81.gtf file obtained from ensembl, overlap between genes and raw reads was calculated in ‘union’ mode using the Rsamtools package [13]. Reads were quantile normalized between samples using the preprocessCore package. After sample quantile normalization, RPKM values for each gene were calculated, which were then log transformed ( ln(n + 1) ) to reduce the effect of extreme RPKM values in subsequent statistical tests. The biomaRt R package was used to provide additional gene annotation such as MGI descriptions (using the M. musculus ensembl database GRCm38.p4).

We analyzed the gene expression of the reciprocal F1 populations with respect to differentially expressed genes, pathways, and allele specific expression. Testing for differences between the two groups of reciprocal crosses was done using two sided t-tests. Due to limited sample sizes of three animals per F1 group, adjustment for multiple testing was not applied for differential gene expression analysis. The original transcriptome sequencing data for all ten animals tested are available at the GEO database (http://www.ncbi.nlm.nih.gov/geo/).

### Pathway analyses

Genes that were differentially expressed (*p* < 0.05) between the two groups of F1 males with RPKM values > 0.5 in at least one of the F1 groups and gene expression differences of > = 20 % were subjected to overrepresentation analyses in KEGG pathways using innateDB [14] to identify pathways associated with these genes [15]. We used the default hypergeometric algorithm and the Benjamini Hochberg algorithm for multiple testing corrections. Heatmaps were created for selected pathways using the mean gene expression per group.

### Analysis of allele specific expression (ASE)

To assess ASE in the reciprocal F1 crosses, bcftools was used to perform SNPs calling on the entire population (all 10 animals: 2 BFMI, 2 B6N, 3 matBFMI, 3 patBFMI). Detected SNPs underwent a rigorous data quality checks to make sure detected SNPs showed sufficient coverage in the entire population. The individuals from the BFMI and B6N pure lines were analyzed to detect homozygous SNPs that showed different alleles between B6N and BFMI. We demanded that all SNPs found in the four parental strain animals had non-missing data (neither low confidence nor low coverage). SNPs were called using the samtools mpileup tool. Subsequently, the generated bcf file was processed using BCFtools [16] and filtered using the provided vcfutils.pl script for SNPs with a 100 reads minimum for the entire population (over all 10 animals). After using the entire population for SNP calling, we reanalyzed all SNPs per individual using the samtools mpileup tool to obtain DP4 measurements for each F1 individual. SNPs with low confidence (Phred probability threshold < 50) or less than 5 high quality reads in an individual were excluded from further analyses. Allele ratios alt/(ref + alt) were calculated for all remaining SNPs. Additionally, we filtered for consistency between the different individuals from the same F1 cross direction. If the standard deviation of the alt/(ref + alt) ratios across the 3 individuals per F1 group was > 20 %, the SNP was removed due to the fact that the data was not consistent across the individuals.

After filtering for high quality SNPs which are homozygous (but different) between the parental strains, $\chi$^2^ scores were calculated for each individual. $\chi$^2^ scores were adjusted to account for direction of the effect (< 50 % vs. > 50 %) and scores were square root transformed to minimize the impact of a single F1 individual (in the F1 groups) showing a highly significant deviation from the expected 50:50 ratio. Summed scores across all individuals in a F1 cross direction (matBFMI and patBFMI) were calculated to obtain a group based $\chi$^2^ score, which were then subtracted from each other to get a group difference score per SNP. To account for over dispersion in the read counts obtained from the DP4 values, we permuted our group based $\chi$^2^ scores to obtain a 5 % FDR threshold for SNPs that show significant ASE in either the patBFMI or the matBFMI group.

### Analysis of transcription factor binding sites

Differentially expressed genes (*p* < 0.05) were submitted to transcription factor binding site (TFBS) analysis. For each gene we extracted the upstream (2000 bp) and downstream (1000 bp) region around the transcription start using the TxDb.Mmusculus.UCSC.mm10 package from bioconductor [17]. All known mouse TFBS motifs were downloaded from the Jaspar database into R using the MotifDb package [18, 19].

Each gene region of 3000 basepairs was scanned for all estrogen receptor related motifs in the Jasper database using the Biostrings package [20], for each motif we recorded the number of matches within the 3000 bp region (Mmusculus-jolma2013-Esrra-2 -UniPROBE-Esrra.UP00079, -JASPAR_CORE-Esrrb-MA0141.1, -JASPAR_2014-Esrrb-MA0141.2). Additionally, position weight matrices for estrogen receptor alpha (ER), estrogen response element (ERE) and the binding site for glucocorticoid receptor (GR)[21, 22] were applied as well.

Since only one weight matrices (jolma2013-Ar) was available in the Jasper database for androgen response elements (AREs) in mouse, we created position weight matrices for androgen response elements using the motifs from Shaffer et al. for ARE, ARE2, ADR3, IR3 [23]. We used a conservative threshold of 90 % for sensitivity and specificity. A list of the applied position weight matrices is given in Additional file 1: Table S3.

Additionally, we compared the occurrence of TFBS in both differentially and allele specific expressed genes with 1,000 permutations of random sets of genes of the respective same group size.

### Quantitative PCR

RNA from ten male matBFMI and ten male patBFMI was extracted from liver tissue. Total RNA was isolated from liver tissue using the NucleoSpin RNA II kit (Macherey-Nagel, Düren, Germany). One μg of total RNA was reverse-transcribed into cDNA using AccuScript High Fidelity Transcriptase (Stratagene Europe, Amsterdam, Netherlands) and Oligo (dT) primers (Ark Scientific, Sigma-Aldrich, München, Germany). The cDNA integrity was checked in a polymerase chain reaction (PCR). For semi-quantitative real-time PCR 20 ng cDNA was mixed with the qPCR mastermix of the Plus Kit for SYBR Green (Eurogentec, Cologne, Germany) and 300 nM of the primers at a final volume of 10 μl per reaction. The amplification protocol included a 10 min DNA denaturation step at 95 °C, followed by 40 cycles consisting of 20 s at 60 °C and 40 s at 72 °C. Quantification of mRNA transcript amounts was performed using the Applied Biosystems® ViiA™ 7 Real-Time PCR System and ViiA™ Software v.1.2.1. Primers for target genes Ar, Peg3 and Srebf1 were designed using Primer3web [24]. Sequences for reference gene primers (Actb, Mrpl46, Srp72) were taken from Hruz et al. [25]. The geometric mean of the three reference genes was used to normalize target gene expression. Relative quantification of gene expression was performed according to ABI's user guide [26]. Further details are provided in Additional file 2: Table S4.

## Results and discussion

### Phenotypes

**Fig. 1:**
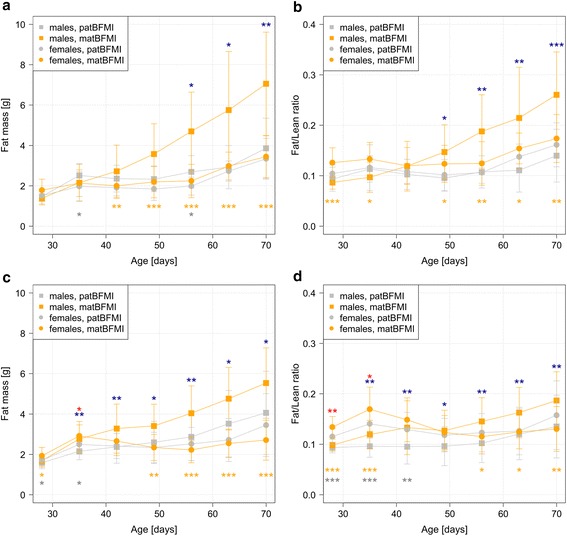
Development of phenotypes of males and females on a high fat diet of the two reciprocal F1-crosses until 10 weeks. The figures show means ± standard deviations of the total fat mass and the total fat mass-to-total-lean mass-ratios in F1-animals without (**a** and **b**, respectively) and with cross-fostering (**c** and **d**, respectively). Paternal and maternal BFMI (pat, mat) indicates direction of cross. Asterisks indicate significant differences between different reciprocal crosses for males (blue) and females (red) and significant differences within the same reciprocal cross between males and females of matBFMI (orange) and patBFMI (grey) (***p < 0.001, **p < 0.01, *p < 0.05)

Since BFMI animals carry a major recessive mutation leading to juvenile obesity in homozygous carriers [27], we expected lean F1-offspring in either cross direction. However, data provided evidence for the development of obesity in F1 males, if the mother originates from the obese BFMI line (matBFMI), while females of the same cross direction and both sexes of the reciprocal cross had normal body weight and fat mass (Table 1). Body mass did not differ significantly between offspring of the reciprocal crosses, but F1 males from BFMI mothers (matBFMI) had 1.83 times more adipose tissue mass at 10 weeks (*p* = 0.0037) than F1 males from BFMI fathers (patBFMI). Since the lean mass was only marginally lower in the obese matBFMI versus the lean patBFMI F1 males (0.98 fold, *p* = 0.054), the fat to lean mass ratio was also 1.86 times higher in matBFMI males (*p* = 0.0009). Moreover, matBFMI also showed ectopic fat storage in the liver with fat content being 1.30 times higher than in patBFMI males from the reciprocal cross (*p* = 0.084). The higher fat mass in matBFMI males began at puberty and was significant from seven weeks on (Fig. 1).

**Table 1:** Phenotypes of 10 weeks old males and females of reciprocal crosses between BFMI and B6N with and without cross-fostering on high fat diet

Phenotypes^1^	F1(BFMI x B6N)		F1(B6N x BFMI)		*p*-Value for direction-of-cross effect	
	BFMI father (patBFMI)		BFMI mother (matBFMI)			
	Males	Females	Males	Females	Males	Females
Maternal fostering						
Body mass (g)	34.23 (3.26)	26.16 (2.02)	36.82 (3.96)	25.27 (2.06)	n.s.	n.s.
Fat mass (g)	3.86 (1.49)	3.34 (1.01)	7.06 (2.56)	3.44 (1.04)	0.0037	n.s.
Lean mass (g)	27.54 (2.06)	20.45 (1.00)	26.93 (2.10)	19.68 (1.09)	0.0536	0.0855
Fat / Lean mass ratio	0.14 (0.05)	0.16 (0.04)	0.26 (0.08)	0.17 (0.05)	0.0009	n.s.
Liver mass (g)	1.55 (0.23)	1.21 (0.10)	1.68 (0.18)	1.16 (0.12)	n.s.	n.s.
Liver triglycerides/ protein (μg/μg)	0.45 (0.09)	0.47 (0.11)	0.59 (0.22)	0.45 (0.08)	0.0843	n.s.
Blood glucose levels (mg/dl)	165.33 (27.20)	135.92 (12.22)	174.25 (23.80)	133.25 (10.71)	n.s.	n.s.
Cross-fostering						
Body mass (g)	34.27 (3.41)	25.96 (3.75)	35.63 (2.65)	24.36 (1.71)	n.s.	n.s.
Fat mass (g)	4.06 (2.06)	3.45 (1.56)	5.54 (1.74) *	2.70 (0.99)	0.0142	n.s.
Lean mass (g)	29.54 (1.84) **	21.69 (3.06)	29.61 (1.68) **	20.64 (1.19) °	n.s.	n.s.
Fat / Lean mass ratio	0.14 (0.06)	0.16 (0.07)	0.19 (0.06) **	0.13 (0.04) °	0.0067	n.s.
Liver mass (g)	1.66 (0.21)	1.22 (0.23)	1.75 (0.15)	1.17 (0.13)	n.s.	n.s.
Liver triglycerides/ protein (μg/μg)	0.15 (0.09)**	0.34 (0.17)°	0.21 (0.08)**	0.26 (0.13)**	0.0307	n.s.
Blood glucose levels (mg/dl)	137.82 (19.07) **	130.36 (13.13)	151.83 (22.7) *	139.29 (20.64)	0.0307	n.s.

^1^shown are means ± standard deviation after correction for litter size, except for blood glucose and liver triglycerides, which were not affected by the litter size. Asterisks additionally indicate significant effects of cross fostering on phenotypes comparing animals of the same direction of cross (patBFMI vs. cross-fostered patBFMI, matBFMI vs. cross-fostered matBFMI) (**p < 0.01, *p < 0.05, °*p* < 0.1)

A cross-fostering experiment was performed to assess the influence of postnatal maternal effects such as milk energy content and behavior during the suckling period on later development of fat deposition in offspring. This experiment validated the results obtained before, but fat deposition was a little lower (Fig. 1). Cross-fostering led to a reduction of adipose tissue mass in male and female F1 offspring of BFMI mothers by approximately 22 % if they were raised by lean B6N foster mothers. Cross-fostered F1 males of BFMI mothers still developed 1.36 times higher fat mass (*p* = 0.0142), had a 1.36 times higher fat to lean ratio (*p* = 0.0067), 1.40 times higher hepatic triglyceride levels (*p* = 0.03070) and higher blood glucose levels (1.10 fold, *p* = 0.03073) than cross-fostered F1 males of the reciprocal cross (Table 2). These data support an effect of the suckling period on later obesity and diabetes development that has been shown before [28–30].

**Table 2:** KEGG pathways affected by the direction of cross in F1 males (*p* < 0.05)

KEGG ID	Major category/Subcategory	Pathway Name	Proportion of genes in pathway	Identified genes ^1^ fold-change (matBFMI vs. patBFMI)	TFBS ^2^	Corrected P-Value for pathway ORA
04710	Organismal Systems/ Environmental adaptation	Circadian rhythm	10 %	Arntl (0.54)	-	0.00087
				Per2 (1.32)	-	
				Per3 (1.71)	-	
04146	Cellular Processes/Transport and catabolism	Peroxisome	5 %	Abcd1 (1.22)	-	0.00094
				Abcd2 (1.52)	-	
				Acsl3 (1.22)	-	
				Crat (1.27)	-	
00830	Metabolism/Metabolism of cofactors and vitamins	Retinol metabolism	5 %	Cyp2c38 (1.61)	Esrra, Esrrb	0.00113
				Cyp2c39 (1.29)	Esrra, Esrrb	
				Cyp4a10 (1.35)	ERE, IR3	
				Cyp4a14 (1.32)	-	
00590	Metabolism/Lipid metabolism	Arachidonic acid metabolism	4 %	Cyp2c38 (1.61)	Esrra, Esrrb	0.00099
				Cyp2c39 (1.29)	Esrra, Esrrb	
				Cyp4a10 (1.35)	ERE, IR3	
				Cyp4a14 (1.32)	-	
00071	Metabolism/Lipid metabolism	Fatty acid degradation	6 %	Acsl3 (1.22)	-	0.00198
				Cyp4a10 (1.35)	ERE, IR3	
				Cyp4a14 (1.32)	-	
00061	Metabolism/Lipid metabolism	Fatty acid biosynthesis	14 %	Acsl3 (1.22)	-	0.00282
				Fasn (1.22)	ERE	
03320	Organismal Systems/ Endocrine system	PPAR signaling pathway	4 %	Acsl3 (1.22)	-	0.00617
				Cyp4a10 (1.35)	ERE, IR3	
				Cyp4a14 (1.32)	-	
00062	Metabolism/ Lipid metabolism	Fatty acid elongation	8 %	Acot4 (1.82)	-	0.00627
				Elovl6 (1.32)	-	
00140	Metabolism/Lipid metabolism	Steroid hormone biosynthesis	4 %	Cyp2c38 (1.61)	Esrra, Esrrb	0.00636
				Cyp2c39 (1.29)	Esrra, Esrrb	
				Cyp7b1 (0.83)	-	
01040	Metabolism/ Lipid metabolism	Biosynthesis of unsaturated fatty acids	8 %	Acot4 (1.82)	-	0.00635
				Elovl6 (1.32)	-	
04270	Organismal Systems/Circulatory system	Vascular smooth muscle contraction	2 %	Avpr1a (0.77)	-	0.01549
				Cyp4a10 (1.35)	ERE, IR3	
				Cyp4a14 (1.32)	-	
02010	Environmental information processing/Membrane transport	ABC transporters	4 %	Abcd1 (1.22)	-	0.01649
				Abcd2 (1.52)	-	
00591	Metabolism/ Lipid metabolism	Linoleic acid metabolism	4 %	Cyp2c38 (1.61)	Esrra, Esrrb	0.01884
				Cyp2c39 (1.29)	Esrra, Esrrb	
04640	Organismal systems/Immune system	Hematopoietic cell lineage	2 %	Dntt (1.24)	-	0.04814
				Il1r1 (0.74)	-	

^1^P-value for differential gene expression between reciprocal F1 groups of males was *p* < 0.05, minimum fold-change difference >20 %, The innateDB tool was used for the KEGG pathway analysis. matBFMI – BFMI mother, patBFMI – BFMI father

^2^Steroid hormone transcription factor binding sites. Esrra/b – estrogen related receptor a/b, ER – estrogen receptor alpha, ERE – estrogen response element, IR3 - androgen inverted repeats of hexameric half-site separated by 3 bp of spacer

### Differential gene expression between F1 males of BFMI mothers and BFMI fathers

**Fig. 2:**
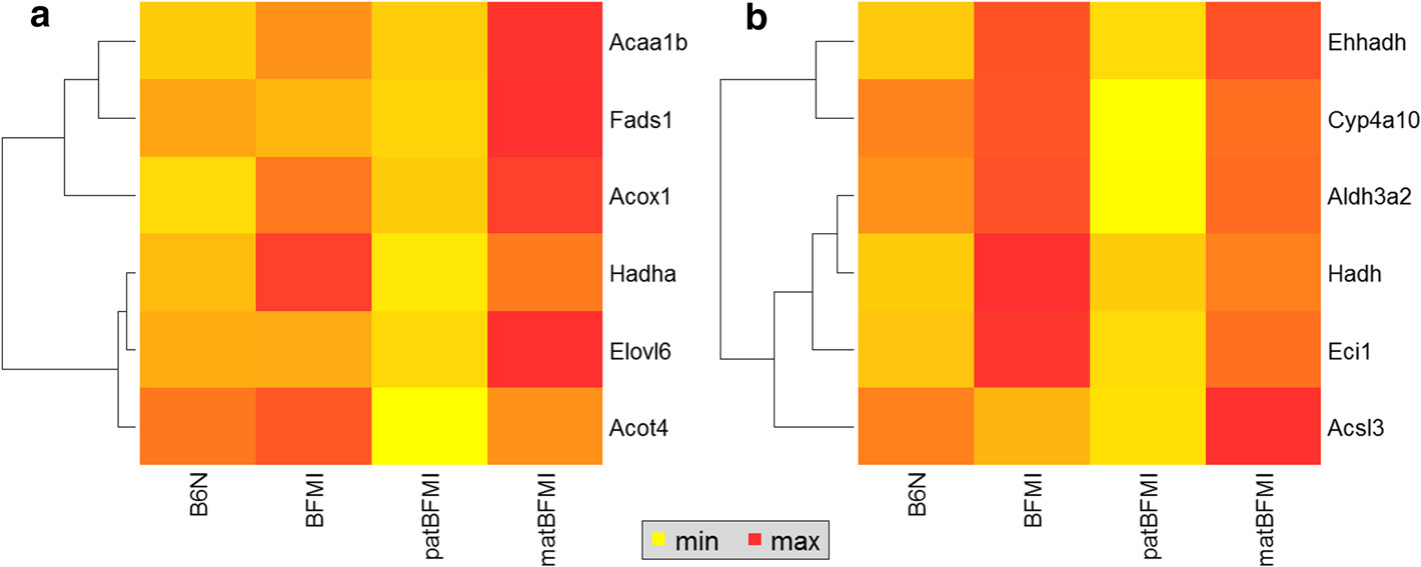
Differential gene expression comparing F1 males from reciprocal crosses and parental strains. Heatmap of genes that were differentially expressed between the reciprocal crosses (p < 0.05) and parental strain gene expression for KEGG pathways of **a**) biosynthesis of unsaturated fatty acids and **b**) fatty acid degradation

Since lipid accumulation of F1 males differed between the reciprocal crosses, we investigated the direction-of-cross effect on hepatic gene expression profiles in males. Overrepresentation analyses of genes that were differentially expressed between the two groups of reciprocal F1 males at *p* < 0.05 and a fold-change difference of at least 20 % provided evidence for alterations of pathways for lipid metabolism, peroxisome, circadian rhythm, cellular transport processes and the endocrine system as a results of the direction of cross (Table 2, Table 3). Most genes with higher expression in the obese F1 males control the cellular utilization of fatty acids for energy production or fat synthesis including the hydrolysis of triglycerides into free fatty acids and glycerol, the conversion of free fatty acids into fatty acyl-CoA esters, the biosynthesis of saturated fatty acids or the degradation and conversion into acetyl-CoA by β-oxidation [31] (Fig. 2). But also genes for the transport between peroxisome and mitochondria via carnitine [32, 33] and the electron carrier generation by the citrate cycle and the respiratory chain in mitochondria were differentially expressed.

**Table 3:** Genes highly differentially expressed between the reciprocal F1 males (*p* < 0.05, fch > 20 %)

Gene Symbol^1^	Chr	Gene description	Mean RPKM^2^		Fch matBFMI/	t Test *P*-value	TFBS^3^
			patBFMI	matBFMI	patBFMI		
**Slc16a7**	10	solute carrier family 16 (monocarboxylic acid transp.), 7	1.91	2.30	1.20	0.0007	Esrrb
Nlrp12	7	NLR family, pyrin domain containing 12	2.66	2.19	0.82	0.0009	ERE
Il1r1	1	interleukin 1 receptor, type I	2.16	1.60	0.74	0.0019	-
St5	7	suppression of tumorigenicity 5	1.97	1.61	0.82	0.0029	-
**Hmgcr**	13	3-hydroxy-3-methylglutaryl-Coenzyme A reductase	3.01	3.98	1.32	0.0034	-
Scara5	14	scavenger receptor class A, member 5 (putative)	1.23	0.55	0.44	0.0050	-
Gpr110	17	G protein-coupled receptor 110	1.48	0.85	0.57	0.0092	-
Grem2	1	gremlin 2 homolog, cysteine knot superfamily	1.50	1.11	0.74	0.0102	-
**Fasn**	11	fatty acid synthase	4.47	5.41	1.21	0.0110	ERE
Selenbp2	3	selenium binding protein 2	5.72	4.76	0.83	0.0113	-
**Cyp4a14**	4	cytochrome P450, family 4, subfam. a, polypept. 14	3.58	4.74	1.32	0.0119	-
**Cyp2c38**	19	cytochrome P450, family 2, subfamily c, polypet. 38	1.46	2.33	1.60	0.0122	Esrra, Esrrb
**Crat**	2	carnitine acetyltransferase	1.79	2.27	1.26	0.0128	-
**Coq10b**	1	coenzyme Q10 homolog B (S. cerevisiae)	1.42	1.98	1.39	0.0138	-
Rai14	15	retinoic acid induced 14	1.98	1.64	0.83	0.0144	-
Mup-ps20	4	major urinary protein, pseudogene 20	1.46	0.51	0.35	0.0145	-
Arntl	7	aryl hydrocarbon receptor nuclear translocator-like	1.90	1.05	0.55	0.0151	-
**Acnat2**	4	acyl-coenzyme A amino acid N-acyltransferase 2	2.37	3.22	1.36	0.0155	Esrrb, IR3
**Abcd2**	15	ATP-binding cassette, sub-family D (ALD), 2	1.14	1.72	1.51	0.0157	-
**Cyp2c39**	19	cytochrome P450, family 2, subfamily c, polypept.39	2.77	3.57	1.29	0.0157	Esrra, Esrrb
Slc45a3	1	solute carrier family 45, member 3	2.15	1.72	0.80	0.0166	-
Sntg2	12	syntrophin, gamma 2	1.75	1.45	0.83	0.0187	-
**Per2**	1	period circadian clock 2	1.26	1.67	1.32	0.0208	-
**Hcn3**	3	hyperpolarization-activated, cyclic nucleotide-gated	0.95	1.51	1.60	0.0208	-
**Gas6**	8	growth arrest specific 6	2.26	2.73	1.21	0.0209	-
Serpina12	12	ser/cys peptidase inhibitor, member 12	2.40	1.94	0.81	0.0211	-
**Elovl6**	3	ELOVL family member 6, elongation of LCFA	3.20	4.17	1.30	0.0214	-
**Acsl3**	1	acyl-CoA synthetase long-chain family member 3	2.24	2.71	1.21	0.0215	-
**Acot4**	12	acyl-CoA thioesterase 4	0.93	1.66	1.79	0.0219	-
**Slc20a1**	2	solute carrier family 20, member 1	1.54	1.87	1.21	0.0219	-
B3galt1	2	UDP beta 1,3-galactosyltransferase, polypept.1	1.52	1.07	0.70	0.0230	-
Cyp7b1	3	cytochrome P450, family 7, subfamily b, polypept. 1	5.11	4.23	0.83	0.0257	-
**Cyp4a10**	4	cytochrome P450, family 4, subfamily a, polypept.10	3.78	5.02	1.33	0.0260	ERE, IR3

^1^Bold gene symbols indicate up regulation in F1 males of a BFMI mother. A list of all differentially expressed genes (*p* < 0.1) is given in Additional file 1: Table S1. Chr – chromosome, matBFMI – BFMI mother, patBFMI – BFMI father, Fch – fold-changes

^2^All values were log-transformed (ln)

^3^TFBS - Transcription factor binding sites for steroid hormones. Esrrb – estrogen related receptor b, ERE – estrogen response element, IR3 – androgen inverted repeats of hexameric half-site separated by 3 bp of spacer

The metabolic changes in obese males were associated with a shift of the circadian rhythm, in which the gene Arntl (0.54 fold, *p* = 0.01), was down- and Per2 (1.32 fold, *p* =0.02) and Per3 (1.71 fold, *p* = 0.03) were up-regulated (Table 2). Disruptions of the normal circadian rhythm have been linked to obesity in Clock [34] and Arntl (alias Bmal1) knock-out mice and feeding experiments [35]. A shift of the circadian rhythm in offspring as a result of *in utero* exposure to maternal obesity has been shown in rats [36].

Indications for impaired β-oxidation in the obese males were found from up-regulated expression of cytoplasmic acetyl-CoA carboxylase (Acaca, 1.20 fold, *p* < 0.06) and citrate lyase (Acly, fold 1.11, *p* < 0.08), which usually inhibits the cytosol-mitochondria transport of the fatty acid derived substrates via allosteric inhibition of Crat (1.27 fold, *p* = 0.01) by malonyl-CoA [37–39]. Furthermore, acyl-CoA thioesterases (Acot) are transporters that regulate the amounts of intermediates from fatty acid oxidation inside mitochondria. The mitochondrial matrix bound Acot2 (1.62 fold, *p* = 0.05), for example, enhances hepatic fatty acid oxidation by its transporter activity to prevent substrate overload [40], which otherwise could impair β-oxidation [41]. In obese F1 males also Acot4 (1.82 fold, *p* < 0.02) and Acot3 (6.49 fold, *p* < 0.08) were up-regulated, which could indicate high amounts of such intermediates inside the mitochondrial matrix, leading to impaired fatty acid oxidation and increased levels of free fatty acids.

Genes encoding peroxisome proliferator-activated receptors (Ppara, Pparg, Ppargc1a, Ppargc1b) that promote β-oxidation and inhibit triglyceride synthesis were not differentially expressed between F1 males of reciprocal crosses. In contrast, the opponent sterol regulatory element–binding factor 1 (Srebf1) which increases fatty acid and triglyceride synthesis [42] was 1.17 fold higher expressed (*p* = 0.03) in the obese F1 males. Many Srebf1 target genes were increased as well (Ar: 2.38 fold, *p* = 0.07; Elovl6: 1.32 fold, *p* = 0.02; Fads1: 1.07 fold, *p* = 0.02; Fasn: 1.21 fold, *p* = 0.01; Gpam: 1.13 fold, *p* = 0.02; Hmgcr: 1.32 fold, *p* = 0.003; Hsd17b7: 1.14 fold, *p* = 0.05; Ldlr: 1.10 fold, *p* = 0.005; Me1: 1.16 fold, *p* = 0.01; Pgd: 1.15 fold, *p* = 0.04). Certain alleles of Srebf1 were found in humans to promote hepatic fibrogenesis [43], which can develop from nonalcoholic fatty liver disease. Consistently with these differences in expression, the hepatic lipase (Lipc) that degrades triglycerides was by trend down-regulated in obese F1 males (0.96 fold, *p* = 0.01).

In a situation, in which fatty acids are preferentially synthesized and accumulated, while their β-oxidation is impaired, the cell has to increase energy generation from alternative fuels. Previous measurements of the respiratory quotient of the BFMI inbred mice provided evidence for a higher use of carbohydrates as the main energy fuel [44]. Succinyl-CoA, for example, is a citrate cycle intermediate that can be produced from degradation of certain amino acids but also from the propanoate pathway. The moderate up-regulation of Mgll (1.17 fold, *p* = 0.01), Aldh3a2 (1.11 fold, *p* = 0.01) and Dak (1.09 fold, *p* = 0.02) from the glycerolipid pathway aims at the conversion of triglycerides to precursor substrates for subsequent glycolysis. Increased carbohydrate metabolism is further supported by up-regulation of the carbohydrate transporters Slc16a7 (1.20 fold, *p* = 0.0007) and Slc16a12 (1.13 fold, *p* = 0.02) in matBFMI (Table 3, Additional file 3: Table S1).

In humans, Slc16a7 catalyzes the rapid transport of lactate, pyruvate, branched-chain oxo acids and other substrates across the plasma membrane of mitochondria and is regulated by estradiol [45, 46]. This regulation could contribute to the fact that female F1 offspring of the BFMI mother are protected against elevated hepatic triglyceride storage.

Among the highest up-regulated genes in matBFMI were the cytochrome P450, family 4, subfamily A members Cyp4a10 (1.35 fold, *p* = 0.03) and Cyp4a14 (1.32 fold, *p* = 0.01), which together with an increase of their rate limiting regulator Por (1.16 fold, *p* = 0.06) is an indicator for increased ω-oxidation [47], a prerequisite for subsequent peroxisomal β-oxidation. These genes were found to play a part in the development of liver steatosis [48, 49]. The cytochrome P450 proteins are involved in the synthesis of cholesterol, steroids, and other lipids. The proteins encoded by Cyp4a10 and Cyp4a14 hydroxylize fatty acids [50, 51]. Recently, fatty acid-hydroxy fatty acids (FAHFAs) have been discovered as a class of lipids with anti-diabetic and anti-inflammatory effects [52]. Higher concentrations of FAHFAs in the obese F1 males due to increased expression of the two Cyp4A genes would be consistent with similar blood glucose levels in male F1 offspring of both cross directions (Table 1).

The hepatic expression of the androgen receptor (Ar) gene was higher in the obese matBFMI F1 males compared to the lean counterparts (2.38 fold, *p* = 0.07). The androgen receptor functions as a steroid-hormone activated transcription factor [53]. Therefore, Ar could contribute to the sex specific obesity effect. The androgen receptor affects the liver metabolism differently in males and females [54]. Besides the liver effects, the androgen receptor protects females against high fat diet-induced obesity and dyslipidemia [55]. Additional studies showed that mice lacking the androgen receptor develop hepatic steatosis and insulin resistance in males but not females under high fat diet [56].

Among the genes that were highly differentially expressed between the two groups of F1 males (*p* < 0.05, fold-change difference min. 20 %, Table 3), six and two genes contain binding sites for estrogen (Acnat2, Cyp2c38, Cyp2c39, Cyp4a10, Fasn, Nlrp12) and androgen (Acnat2, Cyp4a10), respectively. Considering all differentially expressed genes under *p* < 0.05 estrogen (ER), androgen (AR) or glucocorticoid response (GR) elements occur in 220 up- (55 ER, 14 AR, 3 GR) and 189 down-regulated (30 ER, 11 AR, 6 GR) genes in the obese versus lean F1 males (Additional file 3: Table S1).

We compared the occurrence of these transcription factor binding sites in genes being differentially expressed (*p* < 0.05, >20 % fold-change difference) between the obese and the non-obese F1 males with randomly selected genes. We found that binding motifs for estrogen seemed to be under-represented in down-regulated genes (*p* = 0.074). However, when relaxing our restraints and define our down-regulated genes as *p* < 0.05 and do not filter for only the highest fold-changes (> 20 %), this under-representation of estrogen binding sites becomes highly significant (*p* = 0.004) while androgen binding sites tended to be over-represented in up-regulated genes (*p* = 0.056). Therefore, modifications of these genes as a result of epigenetic reprogramming during genetic imprinting or during pregnancy could modify their expression in response to sex-hormone production beginning at puberty.

### Gene expression of hemizygous genetic elements

Differential gene expression on the X and Y chromosome and mitochondria was examined in particular because these homozygous genomic units can be polymorphic between the reciprocal crosses. Among them are the androgen receptor (Ar: 2.38 fold, *p* = 0.07), zinc finger protein X-linked (Zfx: 0.84 fold, p = 0.09) and polyglutamine binding protein 1 (Pqbp1: 1.07 fold, *p* < 0.1), which act as transcription factors. Differentially expressed X chromosomal genes that are involved in the lipid metabolism are ATP-binding cassette, sub-family D (ALD), member 1 (Abcd1: 1.22 fold, *p* = 0.04), HAUS augmin-like complex, subunit 7 (Haus7: 1.21 fold, *p* = 0.05), NAD(P) dependent steroid dehydrogenase-like (Nsdhl: 1.10 fold, *p* = 0.03), monocarboxylic acid transporter (Slc16a2) and glycerol kinase (Gyk) (Table 4, Additional file 3: Table S1). Zfx is well studies in cancer as a proliferation factor; it is also involved in animal size and fertility and lower gene expression was found to inhibit cell growth [57, 58]. Dysfunctional Pqbp1 was associated to lipid metabolism in *C. elegans*. Repression of this gene led to reduction of lipid content in mammalian primary white adipocytes and also triglyceride synthesis from fatty acids was impaired [59]. Since no differences in expression were found for genes on the Y-chromosome, we provide further evidence that the Y chromosome can most likely ruled out as cause for differences in obesity of males. In the class of mitochondrial genes we found mitochondrial NADH dehydrogenase 1 (mt-Nd1) expressed at a lower level (0.95 fold, *p* = 0.02) in obese matBFMI F1 males (Table 4).

**Table 4:** Differentially expressed genes between patBFMI and matBFMI on X chromosome and mitochondria

Gene Symbol^1^	Chr	Gene Description (mgi)	Mean RPKM^2^		Fch matBFMI/ patBFMI	tTest F1	TFBS^3^
			patBFMI	matBFMI			
Trmt2b	X	TRM2 tRNA methyltransferase 2B	2.07	1.83	0.88	0.0008	-
Ssr4	X	signal sequence receptor, delta	3.01	2.84	0.94	0.0013	-
Rps4x	X	ribosomal protein S4, X-linked	4.3	4.17	0.97	0.0026	-
Prdx4	X	peroxiredoxin 4	3.63	3.49	0.96	0.0086	-
Shroom2	X	shroom family member 2	1.66	1.46	0.88	0.0163	-
Rps24-ps3	X	ribosomal protein S24, pseudogene 3	5.17	5.04	0.97	0.0212	-
**Gm5763**	X	predicted pseudogene 5763	0.85	1.52	1.79	0.0255	-
**Nsdhl**	X	NAD(P) dependent steroid dehydrogenase-like	3.15	3.47	1.10	0.0331	-
**Abcd1**	X	ATP-binding cassette, sub-family D (ALD), 1	1.37	1.67	1.22	0.0439	-
Mospd2	X	motile sperm domain containing 2	1.92	1.88	0.98	0.0456	-
**Haus7**	X	HAUS augmin-like complex, subunit 7	1.85	2.23	1.21	0.0486	Esrrb
**Cd99l2**	X	CD99 antigen-like 2	1.77	1.87	1.06	0.0489	Esrra,Esrrb
**Serpina7**	X	serine (or cysteine) peptidase inhibitor, clade A (alpha-1 antiproteinase, antitrypsin), member 7	2.01	2.52	1.25	0.0508	ERE
Pls3	X	plastin 3 (T-isoform)	2.84	2.74	0.96	0.0557	-
Tsc22d3	X	TSC22 domain family, member 3	3.59	3.20	0.89	0.0667	ADR3
Slc16a2	X	solute carrier family 16 (monocarboxylic acid transporters), member 2	2.77	2.62	0.95	0.0668	-
**Ar**	X	androgen receptor	0.26	0.62	2.38	0.0678	-
**Hsd17b10**	X	hydroxysteroid (17-beta) dehydrogenase 10	4.21	4.43	1.05	0.0688	-
Tmsb4x	X	thymosin, beta 4, X chromosome	3.8	3.63	0.96	0.0703	IR3
**Gyk**	X	glycerol kinase	2.9	2.97	1.02	0.0790	Esrra,Esrra, ERE
**Gm14760**	X	predicted gene 14760	3.02	3.18	1.05	0.0803	Esrra,Esrrb
**Wdr13**	X	WD repeat domain 13	1.62	1.7	1.05	0.0831	Esrra, ERE
Gm14373	X	predicted gene 14373	2.49	2.41	0.97	0.0858	-
**Cldn2**	X	claudin 2	3.22	3.45	1.07	0.0870	-
Rlim	X	ring finger protein, LIM domain interacting	2.2	2.12	0.96	0.0910	-
Zfx	X	zinc finger protein X-linked	1.47	1.23	0.84	0.0915	-
**Pqbp1**	X	polyglutamine binding protein 1	2.53	2.70	1.07	0.0971	-
Lamp2	X	lysosomal-associated membrane protein 2	4.14	4.01	0.97	0.0974	Esrra,Esrrb
F8	X	coagulation factor VIII	1.99	1.83	0.92	0.0979	-
Gm6472	X	predicted pseudogene 6472	4.99	4.90	0.98	0.0997	-
mt-Nd1	MT	mitochondrially encoded NADH dehydrogenase 1	6.76	6.42	0.95	0.0165	-

^1^Bold gene symbols indicate up-regulation in matBFMI. Y chromosomal genes were not differentially expressed. Chr – chromosome, matBFMI – BFMI mother, patBFMI – BFMI father, Fch – fold-change

^2^All values were log-transformed (ln)

^3^TFBS - Transcription factor binding sites for steroid hormones. Esrrb – estrogen related receptor b, ERE – estrogen response element, ADR3 – androgen direct repeats of the hexameric half-site, IR3 – androgen inverted repeats of hexameric half-site separated by 3 bp of spacer

### Quantitative PCR validation

We verified our gene expression fold-change results from the RNA-seq analyses performing qPCR for Ar, Peg3 and Srebf1. The qPCR results confirmed the direction of fold-changes in the obese matBFMI animals for the most genes with Ar being 3.27 fold (*p* = 0.004) up-regulated and Peg3 being severely down-regulated (0.36 fold, *p* = 0.002; Additional file 2: Table S4). Srebf1 was found to be 1.66 fold upregulated, although with lower significance (*p* = 0.065) as compared to the RNA-seq results (*p* = 0.027). It is possible that the upregulation of the androgen receptor is a reaction to low androgen levels that are known to be caused by obesity, though a study in cell culture found androgens to inhibit Peg3 expression [60].

### Allele specific expression

Since allele-specific expression could result from parental imprinting, we exploited the sequence information of expressed genes. After rigorous quality check, we found 47,566 SNPs on autosomes (7,470 not in genes, and 40,096 SNPs in 6,260 genes). After selecting SNPs for being different between parental strains BFMI and B6N 26,101 SNPs were left (1,846 not in genes, and 24,255 SNPs in 3,961 genes). After selecting for ASE there were 1,362 SNPs left (139 not in genes, and 1,223 SNPs in 415 genes). Filtering those for concordance yielded 1,315 SNPs (137 not in genes, and 1,178 SNPs in 402 genes, see Additional file 4: Table S2). These SNPs were used to search for parent-of origin-specific expression in the liver of 10 week old males. Deviations from balanced biallelic expression can cause different phenotypes by causing total gene expression differences but also independently since allele functions can be altered due to sequence variants, altering the function of gene products. We found evidence for allelic imbalances for protein coding genes Abca8b, Commd1, H13, Harbi1, Mcts2, Peg3, Plg, Prkd3 and Zrsr1 (Table 5).

**Table 5:** SNPs indicating allelic imbalance in both reciprocal crosses

					Alternative allele^1^ proportion			
Chr	SNP-Position	Ref/Alt	Gene symbol	Gene description	patBFMI	matBFMI	Ratio diff.	TFBS^2^
2	91712576	C/T	Harbi1	harbinger transposase derived 1	48.1	79.7	31.5	Esrra, Esrrb
2	152687856	C/T	Mcts2	malignant T cell amplified sequence 2	88.9	7.2	81.8	-
2	152704657	A/T	H13	histocompatibility 13	18.4	75.9	57.5	-
2	152705549	G/A	H13		7.9	100.0	92.1	
7	6704437	C/T	Peg3	paternally expressed 3 *	93.6	-	-	-
7	6706584	A/G	Peg3		100.0	-	-	
11	22956525	G/A	Commd1	COMM domain containing 1	37.3	57.7	20.3	-
11	22972794	G/T	Commd1		98.8	0.0	98.8	
11	22974882	G/A	Zrsr1	zinc finger (CCCH type), RNA binding motif and serine/arginine rich 1	95.2	4.6	90.7	-
11	109933190	T/C	Abca8b	ATP-binding cassette, sub-family A (ABC1), member 8b	57.6	30.9	26.7	-
11	109933214	C/T	Abca8b		60.8	38.5	22.4	
11	109974591	A/C	Abca8b		67.7	45.7	22.0	
12	104081368	A/G	Serpina4-ps1	serine (or cysteine) peptidase inhibitor, clade A, member 4, pseudogene 1	24.6	60.9	36.3	-
12	104081522	G/A	Serpina4-ps1		27.2	58.0	30.8	
17	12380069	G/T	Plg	plasminogen	22.6	71.7	49.1	-
17	78969687	T/C	Prkd3	protein kinase D3	77.5	57.3	20.2	-

Chr – chromosome, matBFMI – F1 males from BFMI mothers, patBFMI – F1 males from BFMI fathers; * Coverage thresholds for ChiSquare based difference calculations in Peg3 were not met in all matBFMI samples. However, variant calling annotates all matBFMI samples as reference (B6) at these positions, confirming the paternal expression. Further ASE gene details are given in Additional file 2: Table S2

^1^Alternative allele in these cases is the BFMI allele

^2^TFBS - Steroid hormone transcription factor binding sites. Esrra/b – estrogen related receptor a/b, ERE – estrogen response element

Paternal allele expression was identified for paternally expressed 3 (Peg3) and zinc finger (CCCH type), RNA binding motif and serine/arginine rich 1 (Zrsr1). Maternal allele expression was found for the gene H13. Previously, all three genes have been described to be genetically imprinted during gametogenesis and differentially expressed in offspring [61–64]. However, even if parent-of-origin specific expression is proven, the expression level of the paternal allele could differ between offspring due to *cis* or *trans* acting regulation that cannot be derived from RNA-seq data [64]. This was the case for **Peg3** in our study. Interestingly, the obese matBFMI males showed only 0.31 fold expression of Peg3 (*p* = 0.046), which was one of the genes with the most extreme decrease in expression. Peg3 is a Krüppel C2H2-type zinc finger protein, which plays a role in the olfactory system related sexual learning, cell proliferation and p53-mediated apoptosis [65, 66]. In zebrafish, Peg3 inhibits the Wnt pathway [67]. In our data, lower expression of Peg3 in in obese males was accompanied by lower expressions of the Wnt target genes cadherin 1 (Cdh1: 0.75 fold, *p* = 0.03), epidermal growth factor receptor (Egfr: 0.85 fold, *p* = 0.05) and neuropilin 1 (Nrp1: 0.90 fold, *p* = 0.02). High Peg3 expression was recently suggested to protect from high-fat diet induced obesity in F1 offspring of reciprocal crosses between PWK and B6 [68]. The ‘paternal transmission’ of the Peg3 effect was deduced from the observation that B6 develop obesity under high fat diet, whereas PWK mice do not. Our data suggest that different paternal alleles and expression levels of Peg3 act differently in heterozygous BFMI/B6N mice.

Very little is known about the function of the imprinted gene **Zrsr1** although it was associated with the pluripotency state in induced pluripotent stem cells [69]. Furthermore, it could be shown that trans-acting DNA binding proteins, such as methyl-CpG binding domain proteins 2 (Mecp2), methyl-CpG binding domain protein Mbd1 and Mbd3 bind at maternal and paternal Zrsr1 alleles differently [70]. In our F1 male mice, Zrsr1 was 0.80 fold expressed in the obese F1 males (*p* = 0.08). The predominant expression of the maternal **H13** allele was confirmed by our data and the gene was only 0.94 fold expressed (*p* < 0.05) in the matBFMI males.

Differences in expression of imprinted genes could be causal for regulation of down-stream genes contributing to obesity. Recently, paternal intergenerational metabolic reprogramming was found in drosophila, where paternal fat-diet led to changes in the germ cell chromatin state, transcription patterns and modified offspring metabolism. The genetic factors identified in drosophila have been verified in mouse and human studies [71].

We also found a lower proportion of the BFMI **Abca8b** allele expressed by the obese matBFMI males. While the patBFMI expressed between 58–68 %, the matBFMI expressed only 31–46 % of the BFMI allele. This corresponds to a mean difference for the alternative allele proportion of 24 % under a fold-change of 0.87 (*p* = 0.06) in total gene expression. ATP-binding cassette (ABC) transporter genes represent the largest family of transmembrane proteins, which arrange the transport of various molecules across all cell membranes utilizing energy from bound ATP. Most ABC genes move compounds from the cytoplasm to the outside of the cell or into an extracellular compartment (endoplasmic reticulum, mitochondria, peroxisome). ABC transporters shuttle hydrophobic compounds within the cell as part of a metabolic process or outside the cell for transport to other organs or secretion from the body. The Abca subfamily includes the largest Abc genes (several >2000 amino acids) and its members are involved in transport of vitamin A (retinol, etc.) derivatives as well as in disorders of cholesterol transport and high-density lipoproteins (HDL) biosynthesis [72]. Our data from pathway analyzes and ASE indicate that altered functions of ABC transporters (mainly subfamily Abca and Abcd) contribute to the accumulation of body fat in the obese matBFMI males.

## Conclusions

In reciprocal crosses between the obese line BFMI and the lean line B6N, we observed high fat deposition with and without cross-fostering beginning at puberty only in male F1 offspring of obese BFMI mothers, but not in females of the same cross and males and females of the reciprocal cross. Gene expression analysis of liver RNA provided evidence for higher expression of genes controlling fat deposition and impaired β-oxidation of lipids as well as effects on the circadian rhythm in the obese F1 males. The obese F1 males likely use more carbohydrates and amino acids for energy production than lean males. Among the extremely up-regulated genes were Cytochrome A4 genes leading to hydroxylation of fatty acids. Furthermore, typical imprinted genes were differentially expressed between the two groups of males in reciprocal crosses.

The observations led us to the conclusion that parent-of-origin effects during gametogenesis and pregnancy play a significant role for the development of obesity in later life. Postnatal maternal effects significantly influence fat deposition, but could not entirely overwrite perinatal parent-of-origin-effects. Reprogramming of gametes and metabolic imprints during pregnancy set epigenetic marks that lead to differential expression of genes in response to sex-hormone activation beginning at puberty. We suggest that the obese F1 males store more fat due to impaired β-oxidation and cellular compound transport. They compensate the lower energy production from fatty acids by an increased use of alternative fuels for the mitochondrial and non-mitochondrial energy production. The hydroxylases Cyt4a10 and Cyp4a14 are suggested to contribute to increased fatty liver, but the hydroxylated fatty acids are likely nonhazardous. The imprinted genes Peg3, Zrsr1, und H13 and the androgen receptor might play important sex-specific roles for affected down-stream signaling and metabolic pathways. We further suggest that the Y and X chromosomes as well as mitochondria play a minor role in the identified phenotypic differences.

Since the higher adipose tissue mass in F1 males of the BFMI mother was maintained during cross-fostering, parent-of-origin effects of genomic reprogramming during gametogenesis and maternal effects during pregnancy could be causal for the elevated fat accumulation.

Although, we found indicators for substrate inhibition circuits in mitochondria, the hypothesis of impaired substrate selection or mitochondrial substrate overload by fatty acids in BFMI mitochondria needs further research. Furthermore, we like to point out that allele-specific-expression analysis with RNA-seq data requires high control standards. Allele-specific expression could only be tested, if sequence variation was identified between the examined mouse strains. Since the analysis performed in this study was very conservative, the data we present here are highly reliable. Nonetheless, we miss information on genes without variation in the coding sequence and genes with very low transcript levels in liver. Moreover, the molecular mechanisms necessary for allele specific expression in adult tissues need further evaluation. A controlling function of sex hormones in metabolism was shown before and is supported by reduced counts of binding sites for estrogens in down-regulated genes in our data.

## Additional files

Additional file 1:
**Table S3.** Position weight matrices for scanned TFBS. (XLSX 12 kb) Additional file 2:
**Table S4.** Validation of RNA-seq derived fold-changes via qPCR. (XLSX 10 kb) Additional file 3:
**Table S1.** All genes being differentially expressed between both reciprocal crosses (*p* < 0.1). (XLSX 96 kb) Additional file 4:
**Table S2.** Sequence variants measured as monoallelic in one or both reciprocal crosses. (XLSX 814 kb)

## Abbreviations

ASE: allele specific expression
Ar: androgen receptor binding site
ADR3: androgen direct repeats of the hexameric half-site
ARE: androgen response element
B6N: C57BL/6NCrl strain
BFMI: Berlin Fat Mouse Inbred strain 860-12/Hber
Esrra/b: estrogen related receptor a/b
ER: estrogen receptor alpha
ERE: estrogen response element
Fch: fold-change
GR: glucocorticoid receptor
IR3: androgen inverted repeats of hexameric half-site separated by 3 bp of spacer
matBFMI: maternal BFMI F1 animals (male B6N x female BFMI), obese
ORA: overrepresentation analysis (via innateDB)
patBFMI: paternal BFMI F1 animals (male BFMI x female B6N), non-obese
RPKM: Reads Per Kilobase per Million mapped reads
TFBS: transcription factor bindings site
