# Erratum to: ‘The direction of cross affects obesity after puberty in male but not female offspring’

**DOI:** 10.1186/s12864-015-2204-y

**Published:** 2015-11-30

**Authors:** Stefan Kärst, Danny Arends, Sebastian Heise, Jan Trost, Marie-Laure Yaspo, Vyacheslav Amstislavskiy, Thomas Risch, Hans Lehrach, Gudrun A. Brockmann

**Affiliations:** Albrecht Daniel Thaer-Institut für Agrar- und Gartenbauwissenschaften, Humboldt-Universität zu Berlin, Invalidenstraße 42, Berlin, D-10115 Germany; Max Planck Institute for Molecular Genetics, Gene Regulation and Systems Biology of Cancer, Ihnestraße 63-73, Berlin, 14195 Germany

Unfortunately, the original version of this article [1] contained an error. The title was written incorrectly ‘The direction of cross obesity after puberty in male but not female offspring’. The title has been corrected in the original article and is also included correctly above.

## References

[1] Kärst S, Arends D, Heis S, Trost J, Yaspo ML, Amstislavskiy V (2015). The direction of cross affects obesity after puberty in male but not female offspring. BMC Genomics.

